# Reimagining pain procedural education through spatial education co-presence through recorded avatar (SPECTRA) lessons

**DOI:** 10.1016/j.inpm.2026.100822

**Published:** 2026-09-12

**Authors:** Oranicha Jumreornvong, Ana Portilla, Jeniffer Rios Rojas, Rebecca Howard, Mina Girgis, John Rubin, Robert White, Rohan Jotwani

**Affiliations:** a Department of Anesthesia, Critical Care and Pain Medicine, Massachusetts General Brigham, Harvard Medical School, Boston, MA, USA; b Department of Physical Medicine and Rehabilitation, University of Minnesota Medical School, Minneapolis, MN, USA; c Department of Physical Medicine and Rehabilitation, Icahn School of Medicine at Mount Sinai, Mount Sinai Health System, New York, NY, USA; d Department of Anesthesiology, Weill Cornell Medicine, NewYork-Presbyterian Hospital, New York, NY, USA

## 1 Introduction

For more than a century, procedural medicine has relied on a consistent educational paradigm. Trainees acquire technical expertise by standing beside experienced clinicians, observing procedures, assisting under supervision, and gradually progressing toward independent practice. This apprenticeship model, often summarized by the familiar phrase "see one, do one, teach one,” has served generations of physicians well. Nevertheless, despite extraordinary advances in medical technology, the methodology of procedural education has remained largely unchanged (see Table 1).

**Table 1:** Potential advantages and limitations of immersive technologies in procedural education.

Potential advantages	Potential limitations
Preservation of spatial relationships and procedural context	Uncertain transfer to live procedural performance
Repeatable, learner-paced exposure	Repetition without feedback is not deliberate practice
Expanded access to expert instruction	Hardware costs and unequal technological access
Scalable, asynchronous learning	Technical and ergonomic barriers
Multilingual and AI-assisted adaptive learning	Data privacy and governance concerns
Potential for objective competency assessment	Reduced human interaction and mentorship

Today's learners face unprecedented challenges. Procedural interventions continue to grow in complexity, while training duration, case availability, and access to expert mentorship remain limited and vary considerably across institutions and geographic settings [1]. [2] These limitations are particularly relevant in interventional pain medicine. Nearly half of pain fellows report unmet training needs in spinal cord stimulation (SCS), with sufficient case volume, lack of a formal curriculum, and limited faculty expertise among the most commonly cited barriers [3]. These training gaps are particularly relevant given evidence of an association between procedural volume and outcomes in SCS practice [4].Educational initiatives have consequently been developed to address identified gaps in neuromodulation training [5]. Similar variability has been reported in regional anesthesia, where graduating residents demonstrate differences in procedural exposure and confidence in performing peripheral nerve blocks [6]. Together, these findings highlight a persistent challenge in procedural education: providing trainees with consistent access to adequate procedural exposure and educational resources.

Digital technologies have attempted to bridge these gaps. Recorded lectures, surgical videos, virtual simulators, and online procedural libraries have expanded educational access beyond traditional clinical training environments [7]. Simulation approaches range from haptic systems that reproduce tactile aspects of procedures to virtual platforms for spatial and image-guided procedural training [8,9].Immersive virtual reality (VR) further enables three-dimensional, spatially contextualized learning. Although knowledge gains may be comparable to traditional methods, immersive technologies may improve learner engagement, satisfaction, and self-efficacy [10,11].Together, these modalities offer complementary approaches to procedural education, each with distinct educational advantages and limitations.

## 2 From passive observation to shared clinical presence

Procedural learning is inherently contextual. Lave and Wenger's situated learning theory emphasizes that expertise develops through participation in authentic environments, while cognitive apprenticeship highlights modeling, coaching, scaffolding, reflection, and exploration as mechanisms through which expert knowledge becomes accessible to learners [12,13]. These concepts are particularly relevant to procedural medicine, where technical expertise also depends on embodied and multisensory experience.

[14]Immersive technologies may support these principles by providing three-dimensional, spatially contextualized environments for procedural learning [15].Traditional video captures only fragments of the procedural experience, positioning the learner outside the procedural field. In contrast, mixed reality and spatial computing can provide three-dimensional environments that preserve spatial relationships, operator positioning, and procedural context [16]. Immersive environments may also create a sense of embodiment and presence that distinguishes them from conventional two-dimensional instructional media [17]. However, immersion alone does not guarantee active learning; the educational value of these technologies ultimately depends on how learners interact with the virtual environment.

Spatial technologies can preserve depth perception, operator positioning, instrument trajectories, and environmental context that may be difficult to convey through conventional two-dimensional recordings [18]. This distinction parallels the concepts of narratology and ludology in game studies. Narratology emphasizes observation of a predetermined sequence, whereas ludology centers on active participation, decision-making, and interaction within a rule-based environment. Applied to procedural education, VR has the potential to incorporate ludological elements by allowing learners to engage with spatial relationships and procedural decisions. However, VR is not inherently interactive. For example, our motion-capture neuraxial masterclass allows learner to observe a procedural sequence in three-dimensional space but does not require them to make independent procedural decisions. Evidence from surgical and healthcare education suggests that immersive and extended-reality approaches may offer educational advantages over conventional didactic methods, particularly when three-dimensional visualization and spatial understanding are important [19,20].

## 3 Preserving the nuances of procedural expertise

Procedural competence depends on more than memorizing a sequence of steps. Motor performance continuously adapts to real-time perceptual and contextual cues rather than relying on fixed motor plans [21].

Experienced proceduralists recognize anatomical variations, anticipate complications, adjust instrument trajectories, optimize ergonomics, and integrate multimodal information in real time. These behaviors reflect tacit knowledge that can be difficult to convey through textbooks or narrated videos [22]. Procedural expertise is also embodied, incorporating spatial orientation and a learned “feel” for instrument handling that develops through experience [14]. Cognitive apprenticeship addresses this challenge by emphasizing the externalization of expert knowledge so that novices can observe and progressively internalize it.

[23]Spatial recording may preserve elements of procedural expertise that are difficult to capture through conventional media. By simultaneously capturing clinician movement, procedural workflow, equipment positioning, fluoroscopic orientation, and spatial relationships, immersive environments may provide learners with a richer representation of procedural execution. This approach may help bridge the gap between the multisensory nature of clinical practice and the predominantly visual-verbal modalities of conventional teaching [24].

Repeated exposure is another potential advantage of immersive procedural education. Unlike live clinical experiences, spatial recordings can be revisited, allowing learners to review difficult procedural segments and spatial relationships at their own pace. Repetition, however, should not be equated with deliberate practice, which requires structured practice paired with targeted feedback [25]. [26] This distinction is reflected in the Learn, See, Practice, Prove, Do, Maintain framework for procedural skill training, in which immersive technologies may particularly support the “See” and “Practice” phases [27]. As currently constituted, SPECTRA does not provide the feedback necessary for deliberate practice; repeated rehearsal without feedback may reinforce rather than correct errors. Future integration of automated or instructor-based feedback could help address this limitation.

## 4 The emergence of asynchronous Co-presence

Immersive technologies are increasingly being applied to interventional pain education. VR simulators for spinal cord stimulation have demonstrated improvements in trainee confidence, efficiency, and procedural performance [28,29]. However, many collaborative mixed-reality platforms require experts and learners to participate simultaneously, preserving some of the scheduling constraints inherent to traditional apprenticeship [30]. Although expert-learner interaction remains an important component of immersive education, accessibility and equity challenges may limit widespread implementation [31]. Asynchronous co-presence offers a potential alternative by allowing an expert's procedural performance to persist as a spatial recording that learners can access independently.

**SPECTRA** (Spatial Education Co-Presence Through Recorded Avatar Lessons) introduces a framework in which expert procedural performance can be recorded as a persistent three-dimensional educational experience. Rather than requiring instructors and trainees to meet in virtual spaces in real time, an educator's procedural performance can be preserved as a spatial resource that learners may revisit regardless of geographic location or time zone. It records the instructor's movements, gestures, instrument handling, and audio within a shared three-dimensional anatomical environment. Unlike conventional two-dimensional video, positional data as hand position, tool trajectory, and gaze direction can be registered to the underlying anatomical model. During playback, learners observed a recorded avatar of the instructor within the same volumetric environment rather than from a fixed camera perspective. Learners can change their viewpoint, pause to examine instrument position relative to anatomy, and access narration in additional languages without requiring the session to be re-recorded.

In this model, an experienced interventional pain physician performing a fluoroscopically guided procedure is no longer limited to teaching learners who are present during a single case. The recorded experience can be reused while preserving contextual features that may be lost in conventional video. This approach aligns with the cognitive apprenticeship principle of modeling by making expert performance and aspects of procedural reasoning visible to learners, while reducing the temporal constraints that limit scalability [32,33].

### 4.1 Case example: gasserian ganglion intervention module

To illustrate this approach, we developed a module on percutaneous Gasserian ganglion intervention at Weill Cornell Medicine using the Medical Holodeck anatomy sandbox, which incorporates cadaveric dissection data and volumetric imaging of the facial and skull base region [34]. An experienced interventional pain faculty member performed the procedure within the virtual environment while SPECTRA captured their hand position, needle trajectory relative to the foramen ovale, C-arm positioning, and narrated commentary as a spatial data registered to the anatomical model. The module was subsequently deployed to more than 100 medical trainees at Universidad Francisco Marroquín in Guatemala, without on-site U.S. faculty supervision.

*[An excerpt of the recorded module is presented here, illustrating:* the instructor's original approach angle and hand position during needle advancement toward the foramen ovale*].*

### 4.2 Observations from deployment

In the Guatemala deployment, learners without direct access to subspecialty faculty could review spatial relationships relevant to Gasserian ganglion access, including needle trajectory relative to the pterygopalatine fossa and operator positioning within the procedural environment. The asynchronous format allowed these spatial relationships to be reviewed repeatedly and at the learner's own pace.

Formal evaluation of this approach is ongoing. Outcomes of interest include procedural knowledge, learner confidence and satisfaction, comprehension of anatomical spatial relationships, technical usability, immersion, and headset tolerability. SPECTRA is intended to complement, rather than replace, in-person procedural training. This deployment illustrates the potential of spatially anchored recordings to convey procedural relationships that may be difficult to represent through conventional two-dimensional video.

## 5 Building a global procedural classroom

Medical education remains unequal across geographic and institutional settings. Training programs in low- and middle-income countries my face limited access to standardized curricula, simulation infrastructure, and experienced procedural mentors [1,2,35]. Digital educational approaches may also perpetuate existing inequities when they are designed without sufficient consideration of local resources, infrastructure, and educational contexts.

[36]Immersive asynchronous education may help expand access to procedural expertise by allowing educational experiences developed at high-volume or specialized centers to be accessed by learners across geographic and institutional boundaries [20,37]. These technologies should complement rather than replace local mentorship and existing educational systems. Artificial intelligence may further support scalability through multilingual translation and adaptation of educational content to local contexts, potentially reducing language barriers to procedural education [38].

## 6 Artificial intelligence and the future of personalized procedural training

The integration of artificial intelligence with immersive technologies may enable more personalized procedural education.AI-powered virtual tutors and adaptive learning systems have demonstrated potential to support individualized medical education at scale [39,40]. Within immersive environments, spatial context may further contribute to embodied learning and the educational experience [41]. The educational value of these technologies must also be considered alongside their cost and implementation requirements [42].Future AI-enabled systems could identify learner errors, provide individualized feedback, recommend targeted practice, and adapt educational content according to learner performance [43]. Within immersive procedural environments, these tools could supplement expert instruction by answering procedural questions, identifying relevant anatomical landmarks, or generating scenarios for targeted rehearsal.

[44]Immersive technologies may also support competency-based medical education (CBME) by capturing objective measures of procedural performance, such as spatial accuracy, decision-making, efficiency, and technical precision. These measures could complement competency-based frameworks that emphasize achievement of defined outcomes rather than procedural volume alone [45,46]. Entrustable professional activities (EPAs), which assess the level of supervision a trainee requires to perform specific clinical tasks, may provide a framework for integrating such performance data into competency-base assessment [45].

## 7 Challenges and responsibilities

The potential benefits of immersive procedural education must be balanced against important limitations. Questions remain regarding long-term knowledge retention, transfer of technical skills to clinical practice, accessibility, hardware costs, data privacy, and integration into existing curricula [47]. Although learners generally report positive experiences with immersive VR, educational outcomes may be comparable to conventional teaching in some settings, and the overall quality of evidence remains limited [11]. Technical and ergonomic barriers, including connectivity, battery life, field of view, and mild physiological side effects may further constrain implementation [20,48]. Rigorous comparative research is therefore needed to determine which learners, procedure, and educational contexts are most likely to benefit from immersive training.

Technology should also augment rather than replace the human relationships central to medical education. Mentorship provides clinical judgment, individualized feedback, professionalism, empathy, and bedside communication cannot be fully reproduced in virtual environments. Studies of surgical trainees’ experiences with XR similarly emphasize the continuing importance of mentorship and collaboration [31]. Immersive technologies should therefore be viewed as complementary tools that expand opportunities for procedural learning while preserving the essential role of expert supervision.

## 8 Conclusion

Advances in communication and simulation have continually expanded how procedural expertise is taught and shared. Immersive technologies may represent the next step in this evolution by extending procedural learning beyond traditional geographic, institutional, and temporal boundaries [9,49].

By preserving spatial context and enabling repeatable educational experiences, VR and related immersive technologies offer new opportunities to complement traditional procedural training. SPECTRA provides one example of how expert procedural performance can be captured as an asynchronous spatial experience, potentially extending access to procedural instruction across distance and time, However, these technologies should augment rather than replace supervised clinical experience, deliberate practice, and expert mentorship.

As procedural medicine continues to evolve, the value of immersive education will ultimately depend not on technological novelty alone, but on its ability to improve meaningful educational outcomes and support the development of safe, competent clinicians.
